# Design of Bio-Optical Transceiver for In Vivo Biomedical Sensor Applications †

**DOI:** 10.3390/s24082584

**Published:** 2024-04-18

**Authors:** Dimitrios Makrakis, Oussama Abderrahmane Dambri, Abdelhakim Senhaji Hafid

**Affiliations:** 1 School of Electrical Engineering and Computer Science, University of Ottawa, Ottawa, ON K1N 6N5, Canada; dmakraki@uottawa.ca; 2 Department of Computer Science and Operations Research, University of Montreal, Montreal, QC H3T 1J4, Canada; abdelhakim.hafid@umontreal.ca

**Keywords:** bio-nanosensor communication, sensor technology, bioluminescence, nano-communication, neuroscience, biocompatibility, biomedical, in vivo monitoring

## Abstract

This paper presents an enhanced version of our previously developed bio-optical transceiver, presenting a significant advancement in nanosensor technology. Using self-assembled polymers, this nanodevice is capable of electron detection while maintaining biocompatibility, an essential feature for in vivo medical biosensors. This enhancement finds significance in the field of infectious disease control, particularly in the early detection of respiratory viruses, including high-threat pathogens such as SARS-CoV-2. The proposed system harnesses bioluminescence by converting electric signaling to visible blue light, effectively opening the path of linking nano-sized mechanisms to larger-scale systems, thereby pushing the boundaries of in vivo biomedical sensing. The performance evaluation of our technology is analytical and is based on the use of Markov chains, through which we assess the bit error probability. The calculated improvements indicate that this technology qualifies as a forerunner in terms of supporting the communication needs of smaller, safer, and more efficient manufactured sensor technologies for in vivo medical applications.

## 1. Introduction

The combination of nanotechnology and tools from synthetic biology has created a new direction of research that has redefined the concept of the Internet of Things (IoT) [1]. While the limited size of NanoThings allows them to be implemented and dispersed in previously impossible to reach locations, their artificial nature can be unsuitable for certain environments such as natural ecosystems. In order to use biological cells and biocompatible materials for sensing, processing, and communicating inside the human body for medical applications, the Internet of Bio-NanoThings (IoBNT) paradigm has been introduced [2]. The IoBNT promises interesting applications in the medical field, such as in-body sensing that collects health-related data for diagnosis. Two methods have been proposed in the literature to design biocompatible IoBNT systems: Diffusion-based Molecular Communication (DbMC) [3,4,5,6,7,8,9,10,11], and polymer-based wired nano-communication [12,13,14,15,16].

The DbMC systems use molecules as information carriers between transmitters and receivers. The thermal fluctuation in the medium allows the molecules to move randomly towards the destination through the process of Brownian motion [3]. The main challenge of DbMC systems is the presence of InterSymbol Interference (ISI), which is caused by the molecules remaining in the medium after previous transmissions [4]. Both passive [5,6,7,8] and active [9,10] solutions have been proposed for the mitigation of ISI. Additionally, DbMC systems suffer from a very limited achievable throughput and high delay [11].

Wired nano-communication is a promising new method that uses self-assembled biopolymers to create nanowires that link the transmitters with the receivers [12]. Instead of using molecules, wired nano-communication systems use electrons as information carriers, which enhances the achievable throughput and reduces the delay [13]. In [14], the author considered using coaxial nanocables terminated by nanomagnets to form self-aligned nanowires in a hybrid wired and wireless nano-communication system for intrabody applications. The proposed method saves the harvested energy using nanocables inside clusters, which leads to higher packet delivery compared to wireless nano-communication systems. Nevertheless, the wireless part of the hybrid system suffers from high path loss caused by molecular absorption. Another study proposed exploiting the ability of bacteria to generate and transfer electrons in order use them as biocompatible nanocables [15]. However, bacteria do not transfer electrons instantly in the same way that real cables do; rather, they absorb them and use chemical reactions to generate other electrons, then transfer them to other bacteria. This discontinuity in electron transmission is responsible for the very low achievable transmission rate of bacterial cables.

In our previous works [12,13], we proposed using self-assembled actin filaments to construct a conductive nanowire. Actin is one of the most abundant and widely studied proteins in our cells, and its filaments are more flexible and easily controllable compared to microtubules and intermediate filaments. One of the main challenges in developing self-assembled wired nano-communication systems is to detect the electrons at the receiver without losing their biocompatibility. In a recent work [16], we proposed a bio-optical transceiver that detects the transmitted electrons through a nanowire, then generates blue light using bioluminescence. The electrons are used to stimulate a Smooth Endoplasmic Reticulum (SER), which secretes calcium ions inside the transceiver. The interaction of Ca^2+^ ions with the photo-protein *Aequorin* triggers a bioluminescent reaction that emits blue light. When the electrical current stops, the calcium channels close and the Ca^2+^ ions are absorbed and stored again in the SER, terminating the generation of blue light. Moreover, we modeled the designed transceiver as an equivalent circuit and derived the analytical expressions of the equivalent circuit’s components. We then calculated the probability of photon emission for each electrical pulse sent, and proposed an Integrate, Sample, and Dump (ISD) receiver.

In this paper, we provide the Decision Feedback (DF) version of this ISD receiver and evaluate it analytically. We explain the process of inserting the SER inside the nanobubble as well as how to reverse the bioluminescent reaction used in the proposed system. The main contributions of this paper are summarized as follows:

1. We provide the decision feedback version of our proposed ISD receiver.
2. We evaluate the decision feedback version of our proposed ISD receiver analytically and compare with the non-feedback version.

The rest of this paper is organized as follows. In Section 2, we provide an in-depth description of the system design. We explain the process of detecting the electrons and converting the transmitted information into light, and describe the proposed ISD receiver. Section 3 provides the system model of the decision feedback version and evaluates it analytically by developing the corresponding MC state model. The analytical expressions of the state-transitional probabilities are derived and the bit error probability (BER) is calculated. The numerical results are presented in Section 4, where the performance of the receiver with and without decision feedback is compared. Finally, we conclude the paper in Section 5.

## 2. System Design

The use of biological material to detect electrons in wired nano-communication systems is a promising solution to overcome the biocompatibility problem. Nature has found an efficient way to detect electrons and use them to store energy through biochemical reactions such as redox [17], photosynthesis [18], and bioluminescence [19]. Inspired by nature, we proposed to use SER and a bioluminescent reaction to detect transmitted electrons and convert them into light [16]. The conversion of electrons into light in wired nano-communication systems facilitates their detection without losing the biocompatibility and provides a technical solution to create a bridge between nano- and macro-scale systems. This bridge can be used in a plethora of applications that cannot be exhausted within one single publication. A possible application of this technology is to identify viral presence in human lungs before the onset of symptoms. Using the Wired Ad hoc NanoNETwork (WANNET) architecture proposed in our previous work [13], the presence of viral infection triggering the polymerization of actin-based nanowires through the network can be exploited by the proposed biosensor to convert the electric fields generated by charged molecules associated with immune activity inside the lungs into blue light. The detection of blue light by a photodetector inside the lungs is easy, as there is no tissue penetration involved. The ability of the biosensor to detect viral presence with precision down to nano-scale marks a critical step forward in preemptive medical strategies, enabling timely intervention and potentially curbing the spread of infections. This advancement underscores the development of monitoring technologies and, prospectively, the evolution of sophisticated treatment methods aimed at mitigating public health crises.

### 2.1. Bio-Optical Transceiver

**Figure 1:**
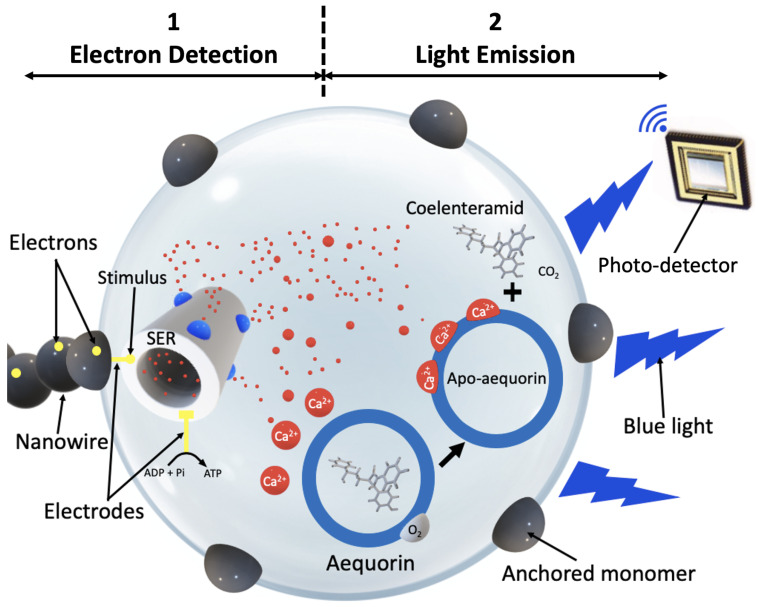
The designed bio-optical transceiver for wired nano-communication networks.

In our recent work, we proposed a bio-optical transceiver. When binary “1” is to be transmitted, electrons are generated during the symbol period and placed on the nanowire. If binary “0” is to be sent, no electrons are placed on the nanowire. The transceiver contains three parts: a part that detects the electrons, a part that emits a blue light and a photo- and information-detection device (see Figure 1). In the first part, the electrons sent through a nanowire and are used to increase the membrane voltage of the SER, resulting in the opening of calcium channels and the release of Ca^2+^ ions. The SER acts as a Ca^2+^ ions storage facility in living cells, and we use it for the same purpose in our system. The extraction of SER from cells is a complex procedure that typically involves cell lysis through homogenization, centrifugation, and identification [20]. Initially, cells are subjected to homogenization to disrupt the cell membrane and release intracellular components without damaging the organelles [21]. The homogenized cell mixture then undergoes centrifugation, a process that separates cellular components based on a component’s size and density. SER fractions are located mostly in the supernatant after centrifugation [22]. Specific biochemical markers are used to separate these fractions from the supernatant. For example, enzyme assays can be used to detect and confirm the presence of SER in collected fractions [23]. This identification step is crucial for ensuring the specificity of the isolation process. When SER has been isolated from cells, its encapsulation into nanospheres is a critical next step. This process requires the use of nanospheres and a suitable encapsulation technique. Polymers are often used to create a core–shell system for the construction of a nanosphere. Polydimethylsiloxane (PDMS) is a commonly used polymer due to its transparency [24] with emulsion polymerization [25]. Endoplasmic reticulum can be encapsulated inside the constructed PDMS hollow nanosphere and used as a store of calcium ions. The capacity of SER to store Ca^2+^ ions is considerable thanks to a buffer called calsequestrin which can bind to around 50 Ca^2+^ cations, thereby decreasing the amount of free Ca^2+^ inside the SER and allowing more calcium to be stored [26].

The number of opened calcium channels is proportional to the intensity of the current stimulating the SER. When the electrical current stops, calcium channels close and the Ca^2+^ ions are absorbed and stored again inside the SER. A picoampere (pA) of electrical current is sufficient to release a micromole of calcium ions [27]. The second part of the transceiver uses the photo-protein *Aequorin*, which emits blue light when binding with Ca^2+^ ions. An advantage of using *Aequorin* is that addition of oxygen molecules is not required, as it already has oxygen bound to it [28], which is not the case with other photo-proteins. Furthermore, it does not involve any diffusible organic factor or direct participation of enzymes, and it can be recycled after use [29]. Oxidation of *Aequorin* is triggered when three Ca^2+^ ions are bound to it, which results in coelenteramid as a byproduct and light emission at a peak wavelength of 470 nm (see Figure 1). To reset and emit light again, *Aequorin* requires replenishment with coelenterazin, which, along with coelenteramid, is nontoxic to humans and safe for use within the body. This replenishment can occur in two ways: by genetically modifying bacteria to secrete coelenterazin continuously, or through direct addition using chemical methods. However, the details of adding coelenterazine are not covered in this paper, under the assumption that it is readily and constantly available in the medium, with its safety for in-body use considered.

### 2.2. ISD Receiver

Every bioluminescent reaction inside the bio-optical transceiver releases over 70 kcal of energy as visible radiation with a wavelength of 470 nm [29]. The intensity of the emitted light is proportional to the released Ca^2+^ concentration during each symbol interval. In the proposed system, the released Ca^2+^ concentration is 0.6 μM. Thus, the detection of information is accomplished by detecting the variation in intensity of the bioluminescent light. If the light energy accumulated during the symbol interval reaches or exceeds a predetermined threshold, then a bit is decoded at the receiver as ‘1’; otherwise, it is decoded as ‘0’. To receive and decode the information transmitted through the variation in light intensity, we proposed an Integrate, Sample, and Dump (ISD) receiver. The studied system is a non-coherent optical system based on the accumulated optical energy at the receiver’s site during a symbol period. When bit 1 is transmitted, an optical waveform $I_{O}\left(t\right)$ is generated:

(1) $$I_{O}\left(t\right)=\bar{I_{O}\left(t\right)}+n_{O}\left(t\right)$$

where $\bar{I_{O}\left(t\right)}$ is the average intensity of the optical waveform at time *t*, which is on the order of tens of ${\mathrm{mW}/\mathrm{mm}}^{2}$. The noise $n_{O}\left(t\right)$ is modeled as AWGN with zero average and variance $N_{O;V}$, while $n_{O}\left(t\right)$ represents the random light intensity fluctuations occurring at time *t*. As its variance is dependent on the strength of $\bar{I_{O}\left(t\right)}$, to simplify the analysis and presentation we consider that the variance is always $N_{O;V}$. The optical signal $O\left(t\right)$ when transmitting *M* bits in a sequence is

(2) $$O\left(t\right)=\sum_{i=1}^{M}b_{i}I_{O}\left(t-((i-1)T\right)),$$

where *T* is the symbol period and $b_{i}$ is the *i*th transmitted bit. We assume a pulse $I_{O}\left(t\right)$ that has a duration of (L+1)T. In the interval [kT,(k+1)T], $O\left(t\right)$ equals

(3) $$\begin{array}{c} O\left(t\right)=b_{k+1}I_{O}\left(t\right)+b_{k}I_{O}\left(t+T\right)+b_{k-1}I_{O}\left(t+2T\right)+\\ \ldots b_{k+1-L}I_{O}\left(t+LT\right)=\sum_{i=0}^{L}b_{k+1-i}I_{O}\left(t+iT\right). \end{array}$$

The optical energy accumulated at the receiver during the period [kT,(K+1)T] equals

(4) $$\begin{array}{c} E_{o}\left(k+1\right)=\int_{kT}^{\left(k+1\right)T}\sum_{i=0}^{L}b_{k+1-i}I_{O}\left(t+iT\right)dt\\ =b_{k+1}\bar{E_{o}\left(0\right)}+E_{ISI}\left(k+1\right)+N_{K+1}^{O}, \end{array}$$

with

(5) $$E_{ISI}\left(k+1\right)=\sum_{i=1}^{L}b_{k+1-i}\bar{E_{o}\left(i\right)}$$

being the optical energy contributed by the InterSymbol Interference (ISI) at the output of the ISD detector collected during the interval [KT,(K+1)T] and $\bar{E_{O}\left(i\right)}=\int_{MT}^{\left(M+1\right)T}\bar{I_{O}\left(t+iT\right)}dt$ the energy that would appear at the output of the ISD detector contributed by the light pulse generated by the transmission of a bit *M* symbol intervals after its transmission interval if the bit is 1. In addition,

(6) $$N_{K+1}^{O}=\sum_{i=0}^{L}b_{k+1-i}\int_{kT}^{\left(k+1\right)T}n_{O}\left(t+iT\right)dt$$

is the noise energy accumulated at the output of the ISD receiver contributed by the random fluctuations of the generated optical intensity. The variance of the noise term $\left(\int_{kT}^{\left(k+1\right)T}n_{o}\left(t+iT\right)dt\right)$ equals $\left(N_{O;V}T\right)$; thus, the variance of $N_{K+1}^{O}$ is $\sigma_{N;O}^{2}\left(K+1\right)=\left(\sum_{i=0}^{L}b_{k+1-i}\right)N_{O;V}T$. It is evident that $\sigma_{N;O}^{2}\left(K+1\right)$ depends on the sequence $[b_{k+1-L,\;}b_{k+2-L},$$\ldots b_{k},\;b_{k+1}]$. Coming back to the accumulated signal strength at the output of the ISD filter, when the transmitted bit $b_{k+1}$ is 1, the accumulated ISI helps the ISD receiver to make correct detection even if only one of the *L* previous bits is 1, as it shifts the collected signal energy at higher values. The ISI becomes problematic when the transmitted symbol $b_{k+1}$ is 0. In addition to the ISI and the self-noise $n_{O}\left(t\right)$ that generates $N_{K+1}^{O}$, there is ambient noise $n_{A}\left(t\right)$ generated by other sources close by (e.g., other similar units) or by ambient radiation. $n_{A}\left(t\right)$ has average $\bar{N_{A}}$ and variance $N_{A;V}$; this generates a noisy signal at the sampled output of the ISD receiver equal to

(7) $$N_{K+1}^{A}=\int_{kT}^{\left(k+1\right)T}n_{A}\left(t\right)dt,$$

which has an average equal to $\bar{E_{A}}=\bar{N_{A}}T$ and variance $\sigma_{N;A}^{2}=N_{A;V}T$. We can model $N_{K+1}^{A}$ as $N_{K+1}^{A}=\bar{E_{A}}+N_{K+1}^{E}$, with $N_{K+1}^{E}$ having an average of zero and variance equal to that of $N_{K+1}^{A}$.

During dark periods, when transmitting bit 0 there is low light intensity generated from incidental light emissions [30]. These emissions are caused by a small number of Ca^2+^ ions which have not been absorbed by SER and continue to interact with *Aequorin* molecules. The power of such light emissions is very small compared to the ambient noise, and is ignored. In the absence of noise, the two energy levels at the output of the ISD receiver are $\bar{E_{O}\left(0\right)}+E_{ISI}\left(k+1\right)+\bar{E_{A}}$ (for $b_{k+1}=1$) and $E_{ISI}\left(k+1\right)+\bar{E_{A}}$ (for $b_{k+1}=0$).

## 3. System Model

### 3.1. Bit Error Probability of the ISD Receiver

Assume absence of ISI, i.e., $E_{ISI}\left(k+1\right)=0$, the optimal location of the threshold in order to make a decision is $\frac{\bar{E_{O}\left(0\right)}}{2}+\bar{E_{A}}$, which is in the middle of the two energy levels shifted upwards by the average power of the ambient noise. The ISD receiver decides as follows:

$$R\left(k+1\right)\overset{{\hat{b}}_{k+1}=1}{\underset{{\hat{b}}_{k+1}=0}{≶}}E_{b}\left(0\right)+\bar{E_{A}}$$

where $E_{b}\left(0\right)=\frac{\bar{E_{O}\left(0\right)}}{2}$. The presence of $E_{ISI}\left(k+1\right)$ shifts all of the energy level upwards or leaves it unchanged, as $\bar{E_{O}\left(i\right)}\geq 0\;\forall \;i$; when $b_{k+1}=1$, the presence of ISI shifts the signal level at $\left[\bar{E_{O}\left(0\right)}+\sum_{i=1}^{L}b_{k+1-i}\bar{E_{O}\left(i\right)}\right]$. In order to produce an error, the summation of the noise terms $\left(N_{K+1}^{O}+N_{K+1}^{E}\right)$ should be $\left(N_{K+1}^{O}+N_{K+1}^{E}\right)\leq -\left[E_{b}\left(0\right)+\sum_{i=1}^{L}b_{k+1-i}\bar{E_{O}\left(i\right)}\right]$. The probability of such an event equals $Q\left(\frac{E_{b}\left(0\right)+\sum_{i=1}^{L}b_{k+1-i}\bar{E_{O}\left(i\right)}}{\sqrt{\left(\left(1+\sum_{i=0}^{L}b_{k+1-i}\right){N_{O;V}+N}_{A;V}\right)T}}\right)$ with $Q\left(x\right)=\frac{1}{\sqrt{2\pi }}\int_{x}^{\infty }e^{-y^{2}}dy$. For $b_{k+1}=0$, the signal level is shifted to $\sum_{i=1}^{L}b_{k+1-i}\bar{E_{O}\left(i\right)}$. There are two scenarios. For $E_{b}\left(0\right)\geq \sum_{i=1}^{L}b_{k+1-i}\bar{E_{O}\left(i\right)}$, in order to produce error, the following should hold: ${(N}_{K+1}^{O}+N_{K+1}^{E})\geq \left[E_{b}\left(0\right)-\sum_{i=1}^{L}b_{k+1-i}\bar{E_{O}\left(i\right)}\right]$, which has probability $Q\left(\frac{E_{b}\left(0\right)-\sum_{i=1}^{L}b_{k+1-i}\bar{E_{O}\left(i\right)}}{\sqrt{\left(\left(\sum_{i=0}^{L}b_{k+1-i}\right){N_{O;V}+N}_{A;V}\right)T}}\right)$. When $E_{b}\left(0\right)<\sum_{i=1}^{L}b_{k+1-i}\bar{E_{O}\left(i\right)}$, error is produced again when ${(N}_{K+1}^{O}+N_{K+1}^{E})>$$\left[E_{b}\left(0\right)-\sum_{i=1}^{L}b_{k+1-i}\bar{E_{O}\left(i\right)}\right]$. In this case, however, there are negative values included, which makes the probability equal to $\left[1-Q\left(\frac{\sum_{i=1}^{L}b_{k+1-i}\bar{E_{O}\left(i\right)}{-E}_{b}\left(0\right)}{\sqrt{\left({{\sum_{i=0}^{L}b_{k+1-i})N}_{O;V}+N}_{A;V}\right)T}}\right)\right]$.

The error probability of the ISD receiver is

(8) $$\begin{array}{cc} P_{e}^{ISD}= & \frac{1}{2^{L+1}}(\sum_{b_{k}=0}^{1}\sum_{b_{k-1}=0}^{1}\sum_{b_{k-2}=0}^{1}\cdots \sum_{b_{k-L}=0}^{1}\\ \; & [Q\left(\frac{E_{b}\left(0\right)+\sum_{i=1}^{L}b_{k-i+1}\bar{E_{o}\left(l\right)}}{\sqrt{\left(\left(1+\sum_{i=0}^{L}b_{k-i+1}\right)N_{O;V}+N_{A;V}\right)T}}\right)\\ \; & +F\left(E_{b}\left(0\right)-\sum_{i=1}^{L}b_{k-i+1}\bar{E_{o}\left(l\right)}\right)\\ \; & +Q\left(\frac{E_{b}\left(0\right)-\sum_{i=1}^{L}b_{k-i+1}\bar{E_{o}\left(l\right)}}{\sqrt{\left(\left(\sum_{i=0}^{L}b_{k-i+1}\right)N_{O;V}+N_{A;V}\right)T}}\right)\\ \; & +F\left(-E_{b}\left(0\right)+\sum_{i=1}^{L}b_{k-i+1}\bar{E_{o}\left(l\right)}\right)\\ \; & +\left(1-Q\left(\frac{\sum_{i=1}^{L}b_{k-i+1}\bar{E_{o}\left(l\right)}-E_{b}\left(0\right)}{\sqrt{\left(\left(\sum_{i=0}^{L}b_{k-i+1}\right)N_{O;V}+N_{A;V}\right)T}}\right)\right)] \end{array}$$

with $F\left(x\right)=\left\{\begin{array}{cc} 0 & \mathrm{for}\;x<0,\\ 1 & \mathrm{for}\;x\geq 0. \end{array}\right.$. For L=1 (only the previous symbol contributes to ISI),

(9) $$\begin{array}{cc} P_{e}^{ISD}= & \frac{1}{2^{2}}(\sum_{b_{k}=0}^{1}[Q\left(\frac{E_{b}\left(0\right)+b_{k}\bar{E_{O}\left(1\right)}}{\sqrt{\left(\left(1+b_{k}\right)N_{O;V}+N_{A;V}\right)T}}\right)\\ \; & +F\left(E_{b}\left(0\right)-b_{k}\bar{E_{o}\left(1\right)}\right)\\ \; & +Q\left(\frac{E_{b}\left(0\right)-b_{k}\bar{E_{o}\left(1\right)}}{\sqrt{\left(b_{k}N_{O;V}+N_{A;V}\right)T}}\right)\\ \; & +F\left(-E_{b}\left(0\right)+b_{k}\bar{E_{o}\left(1\right)}\right)\\ \; & +\left(1-Q\left(\frac{b_{k}\bar{E_{o}\left(1\right)}-E_{b}\left(0\right)}{\sqrt{\left(b_{k}N_{O;V}+N_{A;V}\right)T}}\right)\right)]. \end{array}$$

### 3.2. Decision Feedback Receiver

**Figure 2:**
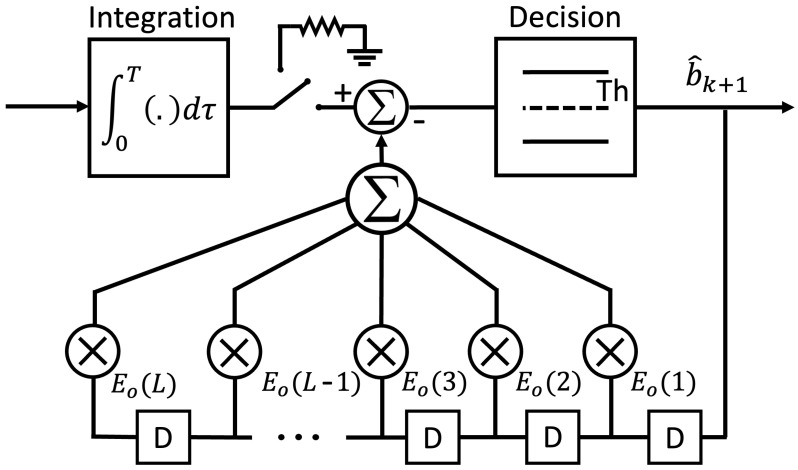
The Decision Feedback version of the proposed ISD receiver; D represents a 1-bit delay storage unit.

Integrate, Sample, and Dump receivers are ideal matched filters for the coherent detection of signals having a rectangular pulse shape and corrupted by AWGN noise [31]; however, as we mentioned earlier, ISI becomes problematic for an ISD receiver when the detected symbol $b_{k+1}$ is 0. The Decision Feedback method is a simple and effective technique that can be used to improve the ISD receiver to better handle ISI. In this paper, we implement the Decision Feedback version of the proposed ISD receiver by adding a digital feedback filter that contains *L* delay storage units of 1 bit, as shown in Figure 2, where *L* is the number of previous symbols contributing to ISI. When a decision is to be made on the *k*th transmitted symbol, the feedback filter forms a weighted linear combination of the previous symbol decisions, cancels the ISI produced by those previous symbols, and removes their presence from the sampled output. Then, a threshold device checks the value after the subtraction and decides the value of the current symbol.

Assuming that there are *L* previous symbols that interfere with the current symbol, the output of the ISD at the sampling moment equals

(10) $$R\left(k+1\right)=b_{k+1}\bar{E_{O}\left(0\right)}+\bar{E_{A}}\;+N_{K+1}^{O}+\;N_{K+1}^{E}.$$

At the time when $b_{k+1}$ is to be decided, $b_{k},b_{k-1},\ldots ,b_{k-L}$ have been decided as ${\hat{b}}_{k},{\hat{b}}_{k-1},\ldots ,$${\hat{b}}_{k-L}$. The new decision metric of the DF receiver is

(11) $$\begin{array}{cc} R_{DF}\left(k+1\right) & =R_{k+1}-\sum_{i=1}^{L}{\hat{b}}_{k-i+1}\bar{E_{o}\left(l\right)}-\bar{E_{A}}\\ = & b_{k+1}\bar{E_{O}\left(0\right)}+\sum_{i=1}^{L}\bar{E_{O}\left(i\right)}\left(b_{k+1-i}-{\hat{b}}_{k+1-i}\right)+\\ \; & \bar{E_{A}}+N_{K+1}^{O}+\;N_{K+1}^{E}. \end{array}$$

If $b_{k+1}=1$ or $b_{k+1}=0$ are transmitted and the noise is absent, the output will be $\bar{E_{O}\left(0\right)}+\sum_{i=1}^{L}b_{k+1-i}\bar{E_{O}\left(i\right)}$ and $\sum_{i=1}^{L}b_{k+1-i}\bar{E_{O}\left(i\right)}$, respectively. If the decisions of $b_{k-i}\left(0\leq i\leq L-1\right)$ are correct, then the decision feedback will eliminate their presence from the sample taken at the output of ISD. What remains is $\bar{E_{O}\left(0\right)}b_{k+1}+\bar{N_{A}}T$, and the threshold is placed at $E_{b}\left(0\right)+\bar{N_{A}}T$. Thus, the new decision law is

$$R_{DF}\left(k+1\right)\overset{{\hat{b}}_{k+1}=1}{\underset{{\hat{b}}_{k+1}=0}{≶}}E_{b}\left(0\right)+\bar{E_{A}}.$$

### 3.3. Markov Chain

**Figure 3:**
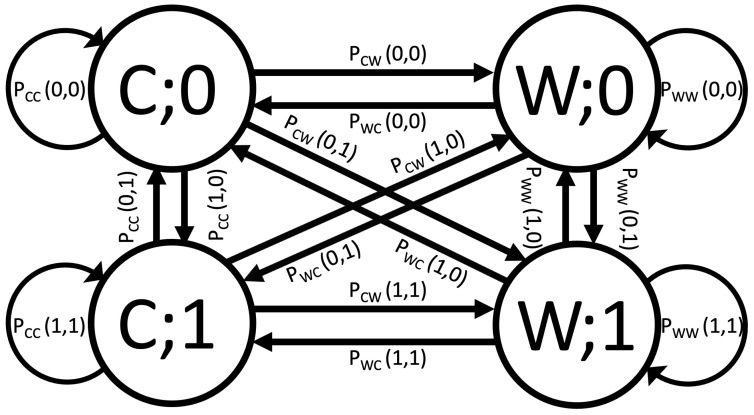
The Markov chain of the studied scenario, where *L* = 1 for the proposed DF receiver.

Our performance analysis, which is based on the use of MCs, includes the option of a decision being incorrect. Because the complexity of the MC model increases exponentially with *L*, we apply the analysis for the case of L=1; however, the approach can be applied to any value of *L*. Figure 3 shows the state diagram of the Markov chain for L=1. The system contains four states: (C;0), (W;0), (C;1), and (W;1). (C;0) means that bit 0 was transmitted and decided to be correct, (W;0) means that 0 was transmitted and was decided to be incorrect, and (C;1) and (W;1) correspond to the same when bit 1 is transmitted. Based on this, we denote the following:

$P_{CX}\left(i,j\right)$: Transition probability from Correct state to (X) state, which can be Correct (X=C) or Wrong (X=W), with *i* and *j* as either 0 or 1. Here, ‘*i*’ is the bit associated with the previous state and ‘*j*’ is the bit associated with the new state.

$P_{WX}\left(i,j\right)$: Transition probability from Wrong state to (X) state; we can further define

$P_{X}\left(a\right)$: Probability of been in the states X;X∈C;W and a∈0;1. The sum of these probabilities is $\sum P_{X}\left(a\right)=1$. The matrix equation describing the MC is

(12) $$\left[\begin{array}{c} P_{C}\left(0\right)\\ P_{C}\left(1\right)\\ P_{W}\left(0\right)\\ P_{W}\left(1\right) \end{array}\right]=\left[\begin{array}{cccc} P_{CC}\left(0,0\right) & P_{WC}\left(0,0\right) & P_{CC}\left(1,0\right) & P_{WC}\left(1,0\right)\\ P_{CW}\left(0,0\right) & P_{WW}\left(0,0\right) & P_{CW}\left(1,0\right) & P_{WW}\left(1,0\right)\\ P_{CC}\left(0,1\right) & P_{WC}\left(0,1\right) & P_{CC}\left(1,1\right) & P_{WC}\left(1,1\right)\\ P_{CW}\left(0,1\right) & P_{WW}\left(0,1\right) & P_{CW}\left(1,1\right) & P_{WW}\left(1,1\right) \end{array}\right]\left[\begin{array}{c} P_{C}\left(0\right)\\ P_{C}\left(1\right)\\ P_{W}\left(0\right)\\ P_{W}\left(1\right) \end{array}\right].$$

We remind the reader that the noise at the output of the ISD receiver at the sampling moment has an average $\bar{N_{A}}T$ and variance ${{\sigma^{2}}_{K+1}^{C}=\left(b_{k+1}+b_{k}\right)N}_{O;V}T+N_{A;V}T$. In the next subsection, we provide the probabilities $P_{CX}\left(i,j\right)$ and $P_{WX}\left(i,j\right)$.

### 3.4. Bit Error Probability of the DF Version

If the decision ${\hat{b}}_{k}$ is correct, then the transition probabilities $P_{CX}\left(i,j\right)$ are

(13) $$\begin{array}{cc} P_{CC}\left(i,j\right) & =P\left(b_{k+1}=j;{\hat{b}}_{k+1}=b_{k+1}|b_{k}=i;\;{\hat{b}}_{k}=b_{k}\right)\\ = & \left(P(i\to j)\right)\left(1-Q\left(\frac{E_{b}\left(0\right)}{\sqrt{\left(\left[b_{k+1}+b_{k}\right]N_{O;V}+N_{A;V}\right)T}}\right)\right), \end{array}$$

(14) $$\begin{array}{cc} P_{CC}\left(i,j\right) & =P\left(b_{k+1}=j;{\hat{b}}_{k+1}\neq b_{k+1}|b_{k}=i;\;{\hat{b}}_{k}=b_{k}\right)\\ \; & =\left(P(i\to j)\right)\left(Q\left(\frac{E_{b}\left(0\right)}{\sqrt{\left(\left[b_{k+1}+b_{k}\right]N_{O;V}+N_{A;V}\right)T}}\right)\right), \end{array}$$

where P(i→j) is the probability that ($b_{k+1}=j$). We assume that the probabilities of $b_{k+1}$ being 1 or 0 are equal; therefore, $P\left(i\to j\right)=\frac{1}{2}$. If the decision ${\hat{b}}_{k}$ is wrong, then the transition probabilities $P_{WX}\left(i,j\right)$ are

(15) $$\begin{array}{cc} P_{WW}\left(i,j\right) & =P\left(b_{k+1}=j;{\hat{b}}_{k+1}\neq b_{k+1}|b_{k}=i;\;{\hat{b}}_{k}\neq b_{k}\right),\\ P_{WC}\left(i,j\right) & =P\left(b_{k+1}=j;{\hat{b}}_{k+1}=b_{k+1}|b_{k}=i;\;{\hat{b}}_{k}\neq b_{k}\right). \end{array}$$

The sampled signal at the output of the receiver after subtracting the ISI is equal to

(16) $$\begin{array}{cc} R_{DF}\left(k+1\right) & =R_{k+1}-{\hat{b}}_{k}\bar{E_{O}\left(1\right)}-\bar{E_{A}}\\ = & b_{k+1}\bar{E_{O}\left(0\right)}+\left[b_{k}-{\hat{b}}_{k}\right]\bar{E_{O}\left(1\right)}+\bar{E_{A}}+N_{K+1}^{O}+\;N_{K+1}^{E}. \end{array}$$

When $\frac{\bar{E_{O}\left(0\right)}}{2}>\bar{E_{O}\left(1\right)}$, the wrong-state transition probabilities are

(17) $$P_{WW}\left(i,j\right)=\frac{1}{2}Q\left(\frac{E_{b}\left(0\right)-\bar{E_{O}\left(1\right)}}{\sqrt{\left(\left[b_{k+1}+b_{k}\right]N_{O;V}+N_{A;V}\right)T}}\right)$$

when $b_{k+1}\neq b_{k}$,

(18) $$P_{WW}\left(i,j\right)=\frac{1}{2}Q\left(\frac{E_{b}\left(0\right)+\bar{E_{O}\left(1\right)}}{\sqrt{\left(\left[b_{k+1}+b_{k}\right]N_{O;V}+N_{A;V}\right)T}}\right)$$

when $b_{k+1}=b_{k}$,

(19) $$P_{WC}\left(i,j\right)=\frac{1}{2}-\frac{1}{2}Q\left(\frac{E_{b}\left(0\right)-\bar{E_{O}\left(1\right)}}{\sqrt{\left(\left[b_{k+1}+b_{k}\right]N_{O;V}+N_{A;V}\right)T}}\right)$$

when $b_{k+1}\neq b_{k}$, and

(20) $$P_{WC}\left(i,j\right)=\frac{1}{2}-\frac{1}{2}Q\left(\frac{E_{b}\left(0\right)+\bar{E_{O}\left(1\right)}}{\sqrt{\left(\left[b_{k+1}+b_{k}\right]N_{O;V}+N_{A;V}\right)T}}\right)$$

when $b_{k+1}=b_{k}$, where $\bar{E_{O}\left(1\right)}$ is the energy per bit for the previous bit.

For the case where $\frac{\bar{E_{O}\left(0\right)}}{2}<\bar{E_{O}\left(1\right)}⟹\frac{\bar{E_{O}\left(0\right)}}{2}-\bar{E_{O}\left(1\right)}\;<0$,

(21) $$P_{WW}\left(i,j\right)=\frac{1}{2}-\frac{1}{2}Q\left(\frac{E_{b}\left(0\right)-\bar{E_{O}\left(1\right)}}{\sqrt{\left(\left[b_{k+1}+b_{k}\right]N_{O;V}+N_{A;V}\right)T}}\right)$$

when $b_{k+1}\neq b_{k}$,

(22) $$P_{WW}\left(i,j\right)=\frac{1}{2}Q\left(\frac{E_{b}\left(0\right)+\bar{E_{O}\left(1\right)}}{\sqrt{\left(\left[b_{k+1}+b_{k}\right]N_{O;V}+N_{A;V}\right)T}}\right)$$

when $b_{k+1}=b_{k}$,

(23) $$P_{WC}\left(i,j\right)=\frac{1}{2}Q\left(\frac{E_{b}\left(0\right)-\bar{E_{O}\left(1\right)}}{\sqrt{\left(\left[b_{k+1}+b_{k}\right]N_{O;V}+N_{A;V}\right)T}}\right)$$

when $b_{k+1}\neq b_{k}$, and

(24) $$P_{WC}\left(i,j\right)=\frac{1}{2}-\frac{1}{2}Q\left(\frac{E_{b}\left(0\right)+\bar{E_{O}\left(1\right)}}{\sqrt{\left(\left[b_{k+1}+b_{k}\right]N_{O;V}+N_{A;V}\right)T}}\right)$$

when $b_{k+1}=b_{k}$.

The error probability is

(25) $$P_{e}^{ISD-DF}=\;P_{W}\left(0\right)+P_{W}\left(1\right).$$

The values of $P_{W}\left(0\right)$ and $P_{W}\left(1\right)$ can be calculated by solving the matrix in Equation (12) and using the condition $P_{W}\left(0\right)+P_{W}\left(1\right)+P_{C}\left(0\right)+P_{C}\left(1\right)=1$. Solving this set of equations by hand is straightforward but cumbersome; instead, we used MATLAB and Mathematica to calculate the final expression of $P_{e}^{ISD-DF}=\;\;\left[Num1-Num2\right]Denom$:

$$\begin{array}{cc} Denom & =P_{CC}\left(0,1\right)-P_{CC}\left(1,1\right)+P_{WC}\left(0,0\right)+P_{WC}\left(0,1\right)\\ \; & -P_{WW}\left(0,0\right)-P_{WW}\left(1,1\right)+P_{CC}\left(0,1\right)\cdot P_{WC}\left(1,0\right)\\ \; & -P_{CC}\left(1,1\right)\cdot P_{WC}\left(0,0\right)+P_{CC}\left(0,1\right)\cdot P_{WC}\left(1,1\right)\\ \; & -P_{CC}\left(1,1\right)\cdot P_{WC}\left(0,1\right)-P_{CC}\left(0,1\right)\cdot P_{WW}\left(0,0\right)\\ \; & +P_{CW}\left(0,1\right)\cdot P_{WC}\left(0,0\right)+P_{CC}\left(1,1\right)\cdot P_{WW}\left(0,0\right)\\ \; & -P_{CW}\left(0,1\right)\cdot P_{WC}\left(1,0\right)-P_{CC}\left(0,1\right)\cdot P_{WW}\left(1,1\right)\\ \; & +P_{CW}\left(1,1\right)\cdot P_{WC}\left(0,1\right)+P_{CC}\left(1,1\right)\cdot P_{WW}\left(1,1\right)\\ \; & -P_{CW}\left(1,1\right)\cdot P_{WC}\left(1,1\right)+P_{WC}\left(0,0\right)\cdot P_{WW}\left(0,1\right)\\ \; & -P_{WC}\left(0,1\right)\cdot P_{WW}\left(0,0\right)-P_{WC}\left(0,0\right)\cdot P_{WW}\left(1,1\right)\\ \; & +P_{WC}\left(0,1\right)\cdot P_{WW}\left(1,0\right)+P_{WW}\left(0,0\right)\cdot P_{WW}\left(1,1\right)\\ \; & -P_{WW}\left(1,0\right)\cdot P_{WW}\left(0,1\right)+P_{CC}\left(0,1\right)\cdot P_{WC}\left(1,0\right)\\ \; & \cdot P_{WW}\left(0,1\right)-P_{CC}\left(0,1\right)\cdot P_{WC}\left(1,1\right)\cdot P_{WW}\left(0,0\right)\\ \; & -P_{CC}\left(1,1\right)\cdot P_{WC}\left(0,0\right)\cdot P_{WW}\left(0,1\right)+P_{CC}\left(1,1\right)\\ \; & \cdot P_{WC}\left(0,1\right)\cdot P_{WW}\left(0,0\right)+P_{CW}\left(0,1\right)\cdot P_{WC}\left(0,0\right)\\ \; & \cdot P_{WC}\left(1,1\right)-P_{CW}\left(0,1\right)\cdot P_{WC}\left(1,0\right)\cdot P_{WC}\left(0,1\right)\\ \; & -P_{CC}\left(0,1\right)\cdot P_{WC}\left(1,0\right)\cdot P_{WW}\left(1,1\right)+P_{CC}\left(0,1\right)\\ \; & \cdot P_{WC}\left(1,1\right)\cdot P_{WW}\left(1,0\right)+P_{CC}\left(1,1\right)\cdot P_{WC}\left(0,0\right)\\ \; & \cdot P_{WW}\left(1,1\right)-P_{CC}\left(1,1\right)\cdot P_{WC}\left(0,1\right)\cdot P_{WW}\left(1,0\right)\\ \; & -P_{CW}\left(1,1\right)\cdot P_{WC}\left(0,0\right)\cdot P_{WC}\left(1,1\right)+P_{CW}\left(1,1\right)\\ \; & \cdot P_{WC}\left(1,0\right)\cdot P_{WC}\left(0,1\right)+P_{CC}\left(0,1\right)\cdot P_{WW}\left(0,0\right)\\ \; & \cdot P_{WW}\left(1,1\right)-P_{CC}\left(0,1\right)\cdot P_{WW}\left(1,0\right)\cdot P_{WW}\left(0,1\right)\\ \; & -P_{CW}\left(0,1\right)\cdot P_{WC}\left(0,0\right)\cdot P_{WW}\left(1,1\right)+P_{CW}\left(0,1\right)\\ \; & \cdot P_{WC}\left(0,1\right)\cdot P_{WW}\left(1,0\right)+P_{CW}\left(1,1\right)\cdot P_{WC}\left(0,0\right)\\ \; & \cdot P_{WW}\left(0,1\right)-P_{CW}\left(1,1\right)\cdot P_{WC}\left(0,1\right)\cdot P_{WW}\left(0,0\right)\\ \; & -P_{CC}\left(1,1\right)\cdot P_{WW}\left(0,0\right)\cdot P_{WW}\left(1,1\right)+P_{CC}\left(1,1\right)\\ \; & \cdot P_{WW}\left(1,0\right)\cdot P_{WW}\left(0,1\right)+P_{CW}\left(0,1\right)\cdot P_{WC}\left(1,0\right)\\ \; & \cdot P_{WW}\left(1,1\right)-P_{CW}\left(0,1\right)\cdot P_{WC}\left(1,1\right)\cdot P_{WW}\left(1,0\right)\\ \; & -P_{CW}\left(1,1\right)\cdot P_{WC}\left(1,0\right)\cdot P_{WW}\left(0,1\right)+P_{CW}\left(1,1\right)\\ \; & \cdot P_{WC}\left(1,1\right)\cdot P_{WW}\left(0,0\right)+1 \end{array}$$

$$\begin{array}{cc} Num1 & =-(P_{CC}\left(0,1\right)-P_{CC}\left(0,1\right)\cdot P_{WW}\left(0,0\right)+P_{CW}\left(0,1\right)\\ \; & \;\cdot P_{WC}\left(0,0\right)-P_{CC}\left(0,1\right)\cdot P_{WW}\left(1,1\right)+P_{CW}\left(1,1\right)\\ \; & \;\cdot P_{WC}\left(0,1\right)+P_{CC}\left(0,1\right)\cdot P_{WW}\left(0,0\right)\cdot P_{WW}\left(1,1\right)\\ \; & \;-P_{CC}\left(0,1\right)\cdot P_{WW}\left(1,0\right)\cdot P_{WW}\left(0,1\right)-P_{CW}\left(0,1\right)\\ \; & \;\cdot P_{WC}\left(0,0\right)\cdot P_{WW}1,1)+P_{CW}\left(0,1\right)\cdot P_{WC}\left(0,1\right)\\ \; & \;\cdot P_{WW}\left(1,0\right)+P_{CW}\left(1,1\right)\cdot P_{WC}\left(0,0\right)\cdot P_{WW}\left(0,1\right)\\ \; & \;-P_{CW}\left(1,1\right)\cdot P_{WC}\left(0,1\right)\cdot P_{WW}\left(0,0\right)) \end{array}$$

$$\begin{array}{cc} Num2 & =(P_{WC}\left(0,1\right)+P_{CC}\left(0,1\right)\cdot P_{WC}\left(1,1\right)-P_{CC}\left(1,1\right)\\ \; & \;\cdot P_{WC}\left(0,1\right)-P_{WC}\left(0,1\right)\cdot P_{WW}\left(0,0\right)+P_{CC}\left(0,1\right)\\ \; & \;\cdot P_{WC}\left(1,0\right)\cdot P_{WW}\left(0,1\right)-P_{CC}\left(0,1\right)\cdot P_{WC}\left(1,1\right)\\ \; & \;\cdot P_{WW}\left(0,0\right)-P_{CC}\left(1,1\right)\cdot P_{WC}\left(0,0\right)\cdot P_{WW}\left(0,1\right)\\ \; & \;+P_{CC}\left(1,1\right)\cdot P_{WC}\left(0,1\right)\cdot P_{WW}\left(0,0\right)+P_{CW}\left(0,1\right)\\ \; & \;\cdot P_{WC}\left(0,0\right)\cdot P_{WC}\left(1,1\right)-P_{CW}\left(0,1\right)\cdot P_{WC}\left(1,0\right)\\ \; & \;\cdot P_{WC}\left(0,1\right)). \end{array}$$

## 4. Numerical Results

This paper introduces the enhanced version of the proposed ISD receiver, which, to the best of our knowledge, marks the first integration of a non-coherent optical system with a biological reaction at the nanoscale. This unique combination distinguishes our work from traditional approaches and establishes novel operational conditions, rendering direct comparisons with existing systems inappropriate. Instead, in this paper we highlight the enhancements of our design over our previously developed ISD receiver model, clearly outlining the achieved advancements. In our previous study [16], we modeled the electron-detecting part of the bio-optical transceiver as an RC circuit and derived its components’ values analytically. In this study, we use the same parameter values in our analysis as in [26]: the capacitance $C=4.5\times 10^{-5}$ $\mathrm{pF}/\mu m^{2}$ and the resistances $R=5.28\times 10^{9}\;\Omega$, $5.28\times 10^{11}\;\Omega$ and $5.28\times 10^{12}\;\Omega$ for 1000, 100, and 10 calcium channels, respectively. The concentration of the released Ca^2+^ ions occurring during each symbol interval, which represents the voltage across the capacitor in the RC circuit, was obtained numerically using Simulink in MATLAB. In our analysis, the symbol interval is T=10μs and the ambient noise is considered to be the dominant noise; thus, the fluctuation noise is ignored $\left(N_{O;V}=0\right)$.

### Bit Error Probability

The system under analysis corresponds to the Optical Shift Keying (OSK) modulation technique. In standard systems, $E_{b}/N_{o}$ represents energy with units of Joules (Watts × seconds), while $N_{o}$ has units of Watts/Hz (Watts × sec); thus, their ratio is dimensionless. The same is the case for SNR (signal power divided by noise power). Using the ratio $\frac{E_{b}}{\sqrt{N_{A;V}}}$ introduces a dependency on time $\left(\frac{Watts*sec}{\sqrt{\frac{{Watts}^{2}}{Hz}}}={sec}^{\frac{3}{2}}\right)$. In this study, the SNR is defined as $SNR=\frac{E_{b}}{\bar{\sqrt{N_{A;V}T}}}$, which ensures that the SNR metric remains dimensionless and free from dependencies on the time and extends the symbol period, allowing for easier comparisons.

**Figure 4:**
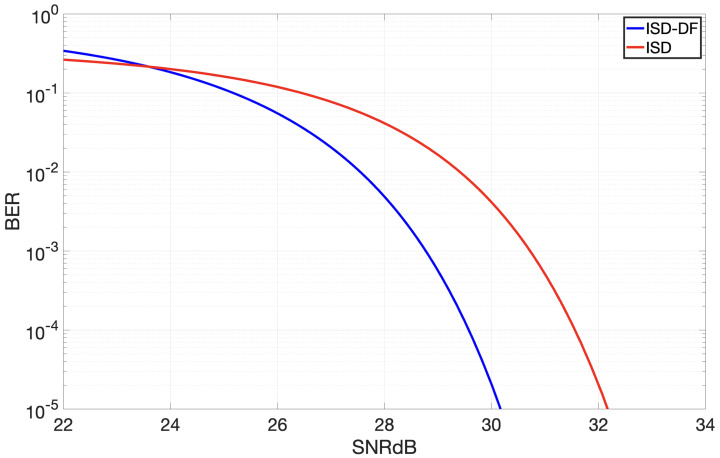
Bit error probability as function of SNR in dB for ISD and DF versions when opening 1000 calcium channels.

The integration of a decision feedback filter augments the proposed receiver’s proficiency in mitigating ISI. This improvement is clearly evidenced by the data presented in Figure 4. In this figure, the BER versus SNR curves are displayed for the ISD receiver without Decision Feedback and the ISD with Decision Feedback (ISD-DF). From this comparison, it is evident that the introduction of Decision Feedback improves the performance of the receiver by 2.06 dB, for a bit error probability of $10^{-4}$.

The probability of bit errors during data transmission depends greatly on the amount of optical power used at the transmitting end. Amplifying the intensity of the light can enhance the system’s overall efficiency. Nevertheless, for in vivo medical use it is essential to strike a balance. Excessively high light intensities can be detrimental to living cells, posing a delicate trade-off between optimizing system efficiency and ensuring cellular safety. Figure 4 shows that the ISD has an SNR of 31.46 dB with an error probability of $10^{-4}$, while the ISD-DF registers an SNR of 29.4 dB. By fixing the value of $N_{A;V}T$ at $6\times 10^{-8}$, it is possible to determine the $E_{b}$ values for the ISD and ISD-DF operating at the same *T* value. Comparing the logarithmic ratio of their $E_{b}$ values, we find that, for the same bit error rate, the ISD-DF consumes about 2.027 dB less energy per bit compared to the ISD. This means that the ISD-DF system operates at a power level that is 60% of the power at which the ISD operates, representing a significant decrease.

**Figure 5:**
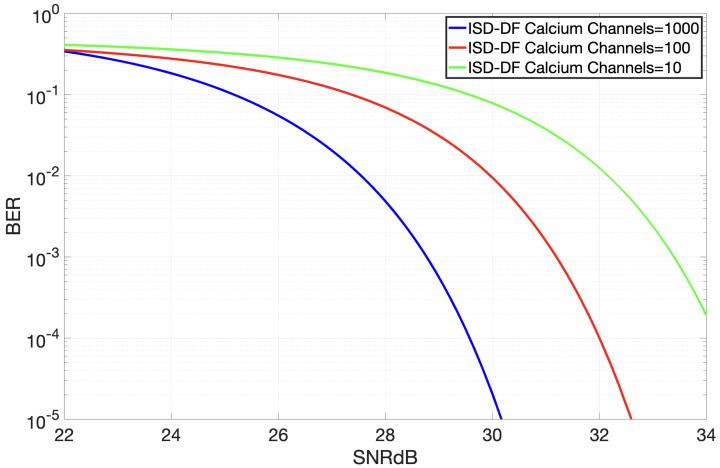
Bit error probability as function of SNR in dB for the DF version when opening different numbers of calcium channels.

Figure 5 provides detailed insight into the performance variations of the improved receiver in response to changes in the number of open calcium channels. Here, a pattern can be observed in that the bit error probability increases as the number of calcium channels decreases; this is because having fewer channels makes signal transmission more challenging, increasing the likelihood of errors. As outlined in our earlier study [16], adding more channels makes the receiver larger, presenting a balancing act between efficiency and miniaturization. Figure 5 shows that the DF version of the proposed receiver, with only 100 channels, has a mere 0.44 dB performance drop compared to the ISD model, which uses 1000 channels. This means that 100 channels are enough for ISD-DF, as further increases yield minimal benefit.

## 5. Conclusions

This study marks a significant step forward in the realm of in vivo biomedical sensing, offering a refined bio-optical transceiver that combines nanotechnology with the vital aspect of biocompatibility. The capacity to identify viral infections with nano-scale precision by harnessing self-assembled polymers and bioluminescence represents a promising application and a major advancement towards the development of smaller, biocompatible in vivo sensors designed for early disease detection. The results of our bit error rate evaluation, conducted by analytical means with the use of Markov chains, confirms the reliability of the proposed system. The advancements detailed in this study pave the way for future innovations in medical sensor technology, promising to enhance patient care and biomedical monitoring with the use of more efficient, smaller, and safer biosensors.

## Data Availability

Data are contained within the article.

## Abbreviations

The following abbreviations are used in this manuscript:

BER	Bit Error Rate
DbMC	Diffusion-based Molecular Communication
DF	Decision Feedback
IoBNT	Internet of BioNanoThings
IoT	Internet of Things
ISD	Integrate, Sample, and Dump
ISI	InterSymbol Interference
MC	Markov Chain
OSK	Optical Shift Keying
PDMS	Polydimethylsiloxane
SER	Smooth Endoplasmic Reticulum
SNR	Signal-to-Noise Ratio
WANNET	Wired Ad hoc NanoNETwork
